# Multifaceted role of CNPY2 beyond ER stress: Disease implications and therapeutic potential

**DOI:** 10.15698/cst2026.03.316

**Published:** 2026-03-06

**Authors:** Shima Ebadollahibaruq, Lingbin Meng, Feng Hong

**Affiliations:** 1 Pelotonia Institute for Immune-Oncology, The Ohio State University Comprehensive Cancer Center, The Ohio State University Wexner Medical Center, 410 W 12th Ave, Columbus, OH, 43210, USA.; 2 Division of Medical Oncology, Department of Internal Medicine, The Ohio State University Wexner Medical Center, 410 W 12th Ave, Columbus, OH, 43210, USA.

**Keywords:** Canopy homolog protein 2, unfolded protein responses, cancer, tumor microenvironment, Endoplasmic reticulum stress, neurodegenerative disease, cardiovascular disease

## Abstract

Canopy homolog protein 2 (CNPY2), an endoplasmic reticulum (ER) luminal protein exhibits broad tissue distribution and regulates cellular homeostasis, including unfolded protein responses (UPR), mitochondrial dynamics, oxidative stress, and apoptosis. Beyond its role in cancer progression through pathways such as NF- κ B, AKT/GSK3 β , PI3K/Akt/mTOR and HIF-1 α , promoting epithelial-mesenchymal transition (EMT), tumor survival and metastasis, CNPY2 is also critical in non-cancer conditions. In neurodegenerative disorders including Parkinson’s and Huntington’s, it exerts neuroprotective role by reducing oxidative stress and mitochondrial dysfunction. In cardiovascular tissues, CNPY2 leads to hypoxia-driven angiogenesis, tissue repair, and ischemia-reperfusion protection. Moreover, recent meta-analyses have linked CNPY2 downregulation with Keratoconus pathogenesis, further highlighting its tissue- specific roles. Hence, this review meticulously dissects CNPY2’s structural characteristics, expression patterns, and biological functions across cancer, cardiovascular disease, inflammation and neurological disorders, emphasizing its role on tumor initiation, microenvironmental stress, and chemoresistance, and evaluating its potential as a therapeutic target.

## INTRODUCTION

Canopy homolog proteins (CNPYs) are a family of ubiquitously expressed small proteins (21–30 kDa) with unique structural characteristics and diverse biological functions. To date, five CNPY family members have been identified in mammals: CNPY1, CNPY2, CNPY3, CNPY4, and CNPY5 1. These proteins composed of 182 to 278 amino acids, share a conserved structural framework characterized by four hydrophobic α -helices and six highly conserved cysteine (cys) residues, that form three characteristic intramolecular disulfide bonds, aligning them with the saposin-like protein (SAPLIP) family 2. Although CNPYs share the saposin fold with SAPLIP proteins, known for their roles in lipid metabolism and surfactant stabilization, the conserved interhelical residues in CNPYs provide distinct structural and functional properties 3.

**Figure 1:**
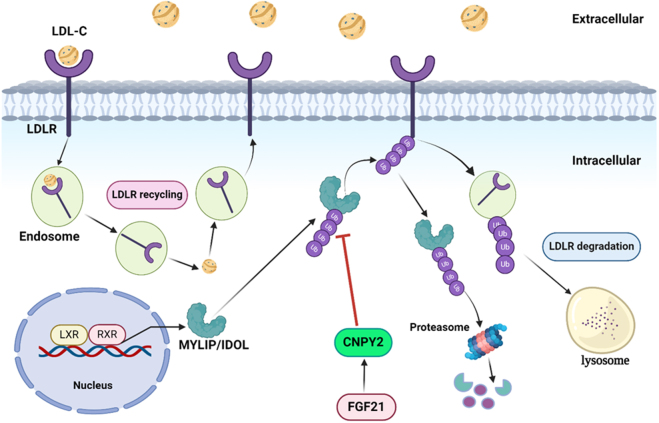
Schematic representation of CNPY2’s role in MYLIP/IDOL-mediated LDLR degradation. CNPY2 regulates the MYLIP/IDOL-mediated degradation of the low-density lipoprotein receptor (LDLR), a key process in cholesterol metabolism. MYLIP/IDOL expression is controlled *via* the liver X receptor-retinoid X receptor (LXR-RXR) heterodimer, which facilitates LDLR ubiquitination and subsequent lysosomal degradation. LXR activation enhances MYLIP expression, leading to improved LDLR lysosomal degradation, decreased LDLR accessibility on the cell surface, and elevated plasma LDL-cholesterol (LDL-C) levels. Conversely, fibroblast growth factor 21 (FGF21)-induced overexpression of CNPY2 suppresses MYLIP/IDOL activity, promotes LDLR expression, and enhances cholesterol clearance. LXR, the liver X receptor; RXR, retinoid X receptor; LDLR, low-density lipoprotein receptor; LDL-C, LDL-cholesterol. Figure created with BioRender.com (https://www.biorender.com/).

All mammalian CNPYs, except the CNPY1, possess N-terminal signal peptides and C-terminal KDEL-type ER-retention sequences (*e.g.*, HDEL in CNPY2, PDEL in CNPY3, PEDL in CNPY4, and REEL in CNPY5), facilitating their localization to the endoplasmic reticulum (ER) 3. These features enable CNPYs to modulate the secretory transport of various proteins and play crucial role in a wide range of biological process 3. In recent years, research on CNPY2, as one of critical ER stress modulators, has expanded remarkably, particularly regarding its involvement in various diseases 4–7. There is a growing need to synthesize and critically evaluate the current knowledge of its biological functions and clinical significance. This review aims to provide a comprehensive analysis of CNPY2’s roles in cancer, cardiovascular diseases, and neurological disorders, with particular emphasis on its molecular mechanisms, interactions within the tumor microenvironment, and its potential as both a biomarker and a therapeutic target.

## CNPY2 STRUCTURE, FUNCTION AND ISOFORMS

Canopy homolog 2 (CNPY2) is notable for its role as an ER luminal protein and is prominently distributed in the liver, heart, and lungs. Beyond its ER localization, CNPY2 is also detected at the plasma membrane, within the intracellular space, and in the perinuclear region, emphasizing its involvement in various physiological and pathological processes 8. It was initially identified in GenBank under various designation, such as HP10390, MIR-interacting saposin-like protein (MSAP), putative secreted protein Zsig9 (ZSIG9), and transmembrane protein 4 (TMEM4) 8, 9.

Human CNPY2 consists of 182 amino acids, with the N-terminal 20 amino acids shaping a signal peptide, suggesting a potential extracellular function. Despite lacking a hydrophobic transmembrane region, CNPY2 possesses a C-terminal HDEL sequence identified by KDEL receptors, ensuring its ER retention and preventing secretion 8. Structurally, CNPY2 comprises four α -helices, four β -strands, and six conserved cysteine residues, that form three disulfide bonds: Cys28-Cys171, Cys31-Cys164, and Cys86-Cys137 3. These disulfide bonds are fundamental for its interaction with the luminal domain of protein kinase RNA-like endoplasmic reticulum kinase (PERK-LD), an integral component of the unfolded protein responses (UPR). In line with this phenomenon, Hong *et al.* have confirmed that mutations disrupting these cysteine residues with alanine (C28A, C31A, C86A, C137A, C164A, C171A) completely disrupt the CNPY2-PERK interaction, highlighting the importance of structural integrity for its function 10.

CNPY2 exists in two isoforms (12): CNPY2 isoform 1 (20.65 kDa), the longer variant, and CNPY2 isoform 2 (9.12 kDa), the shorter variant 11. Both isoforms share a common N-terminal region, including a signal peptide consisting of the first 20 amino acids, but differ in their C-terminal sequences, which affect their localization and function as secreted proteins. Unlike isoform 1, CNPY2 isoform 2 lacks an ER retention signal (HDEL), which may contribute to its distinct biological activities. Both isoforms have been implicated in cancer progression, particularly in colorectal cancer (CRC) 12. CNPY2 isoform 1 has also been associated with broader physiological roles, including lipid metabolism 13 and cardiovascular function 8, 14. Studies have reported that CNPY2 isoform 1 functions as a secreted extracellular protein and is widely expressed across various epithelial tissues 8. In contrast, CNPY2 isoform 2 is commonly detected in primary CRC tumors, liver metastases, surrounding normal tissues, and healthy liver tissue 15.

## CNPY2 AS AN INDIRECT MODULATOR OF LDLR STABILITY

Emerging evidence suggests that CNPY2 is involved in the regulation of protein stability via its interaction with myosin regulatory light chain interacting protein (MYLIP). MYLIP, also known as IDOL (Inducible Degrader of the LDL receptor), functions as E3 ubiquitin ligase. It interacts with the UBE2D family of E2 ubiquitin-conjugating enzymes (UBE2D1-4), promoting the ubiquitination and subsequent degradation of target proteins, including low-density lipoprotein-cholesterol receptor (LDLR) 16–19. Through this mechanism, MYLIP plays a vital role in lipid homeostasis via regulating LDLR turnover.

According to relevant research, CNPY2 regulates lipid metabolism by interacting with fibroblast Growth Factor 21 (FGF21) and impacts on LDLR levels in RAW 264.7 and Huh7 cells 13, 20. FGF21 has been shown to exert dual regulatory effects, upregulating CNPY2 while downregulating MYLIP, which together contributes to increased LDLR levels (Figure 1). On the contrary, CNPY2 knockdown reduces the FGF21-induced elevation of LDLR, underscoring the essential role of CNPY2 in FGF21-regulated lipoprotein metabolism. Furthermore, CNPY2 has been shown to protect very-low density lipoprotein receptor (VLDLR) , apolipoprotein E receptor 2 (Apo-ER2) from lysosomal degradation via inhibiting MYLIP-mediated ubiquitination 13. These findings suggest that CNPY2 is a promising therapeutic target for lipid-related disorders and emphasize its regulatory function in lipoprotein metabolism.

## CROSSTALK BETWEEN CNPY2 AND THE UNFOLDED PROTEIN RESPONSE (UPR)

**Figure 2:**
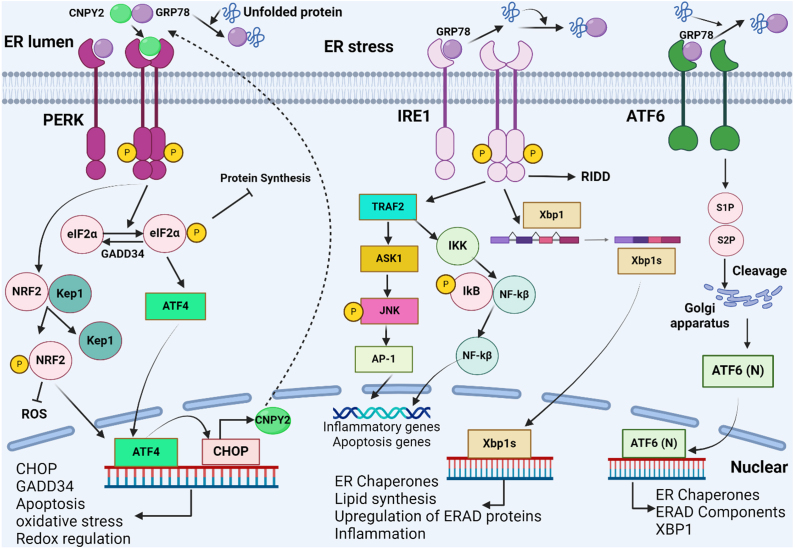
Endoplasmic reticulum stress and the adaptive unfolded protein response (UPR) pathway in cellular homeostasis. Excessive accumulation of unfolded or misfolded proteins in the ER activates three ER stress sensors: IRE1, PERK and ATF6. These pathways promote the expression of chaperones, the synthesis of ER and Golgi structural components, and the ER- associated degradation (ERAD) pathway, improves protein folding and reduces stress-induced cellular damage. Under prolonged stress, the UPR shifts to apoptosis, mediated by IRE1-ASK1-JNK signaling and ATF6 activation. The figure was created with BioRender.com (https://www.biorender.com/).

Findings support the notion that CNPY2 plays a pivotal role in ER stress and UPR. Disruption in ER homeostasis, collectively referred to as “ER-stress”, trigger the UPR, an adaptive mechanism that alleviates the accumulation of unfolded or misfolded proteins 21. Beyond its classical function in maintaining intracellular protein homeostasis, the UPR also governs diverse physiological and pathological responses, including innate immunity, energy metabolism, and cellular differentiation 22, 23. In mammalian cells, the UPR is orchestrated by three ER transmembrane receptors: double-stranded RNA-activated protein kinase-like ER kinase (PERK), inositol-requiring enzyme 1 (IRE1), and activating transcription factor 6 (ATF6), which are remained in an inactive state via forming stable complexes with glucose-regulated protein 78 (GRP78) on the ER membrane 24. Upon ER stress, the accumulation of unfolded or misfolded proteins leads to the dissociation of GRP78 binding, initiating downstream signaling. If unresolved, prolonged ER stress triggers apoptosis (summarized in Figure 2)(1).

## CNPY2 AS A MOLECULAR SWITCH IN PERK-CHOP PATHWAY

Among the three branches of UPR, the CNPY2- PERK-C/EBP homologous protein (CHOP) pathway is a well-established regulatory pathway essential for UPR activation 10, 25. PERK contains an N-terminal luminal domain that interacts with GRP78 and a cytoplasmatic serine/threonine kinase domain that activates downstream signals 26, 27. Upon ER stress, CNPY2 facilitates dissociation of GRP78 from PERK, promoting PERK homodimerization and phosphorylation 10. Activated PERK phosphorylates eukaryotic initiation factor 2 alpha (eIF2 α ) at Ser51, reducing general mRNA translation while allowing selective translation of activating transcription factor 4 (ATF4), which activates ER stress-response genes 28–31. PERK also phosphorylates nuclear factor erythroid 2-related factor 2 (NRF2), leads to its dissociation from Kelch-like ECH-associated protein 1 (KEAP)1, and enabling promoting ATF4 transcriptional activity 32–35.

## CNPY2-CHOP FEEDBACK LOOP AND UPR AMPLIFICATION

Under chronic ER stress, ATF4 amplifies CHOP expression, which shifts the UPR toward apoptotis. CHOP induces GADD34 to restore protein synthesis and suppress PERK signaling as negative feedback. However, sustained CHOP activity promotes apoptosis by regulating BCL-2 family members and increasing reactive oxygen species (ROS) 36. Interestingly, CHOP also transcriptionally upregulates CNPY2, establishing a positive feedback loop that reinforces UPR signaling 10. These findings position CNPY2 as a principal regulator in both adaptive and maladaptive ER stress responses, making it as a promising therapeutic target in chronic UPR-associated diseases such as non-alcoholic fatty liver disease (NAFLD), inflammation, and cancer.

## MULTIFACETED ROLES OF CNPY2 IN CANCER PATHOGENESIS

### Regulation of the androgen receptor *via* CNPY2 in prostate cancer

CNPY2 has also been implicated in the progression of prostate cancer *via* inhibiting MYLIP-mediated degradation of the androgen receptor (AR) protein in prostate cancer cells 37. It is well-established that AR overexpression and AR-regulated signaling pathways are pivotal drivers of prostate cancer progression particularly in castration-resistant prostate cancer (CRPC) 38. In a genetic screen using Drosophila model, CNPY2 expression was found to trigger prostate cancer cell growth and metastasis 39. Under normal physiological situation, MYLIP facilitates AR ubiquitination, targeting it for degradation through the ubiquitin-proteasome pathway 37. However, CNPY2 disrupts this regulatory mechanism by preventing MYLIP’s ubiquitination function, particularly by restraining its interaction with MYLIP-UBE2D1, a key E2 ubiquitin- conjugating enzyme 37. Interestingly, a positive correlation between CNPY2 expression and levels of AR and AR target genes has been observed in human prostate cancer tissues. By suppressing AR degradation, CNPY2 increases prostate cancer cell proliferation and upregulates KLK3 gene expression, the gene encoding the prostate specific antigen (PSA) 37. Future investigation into the CNPY2-MYLIP axis could provide new insights into its role in both cancer and metabolic disorders. Discovering additional MYLIP targets could broaden our understanding of CNPY2’s regulatory functions beyond AR and LDL pathways.

### CNPY2 in LUNG CANCER: Regulation of AKT/GSK3 β and NF- κ B pathways, and chemoresistance

CNPY2 is a vital regulator of non-small-cell lung cancer (NSCLC) progression, influencing tumor growth and therapeutic resistance through distinct molecular pathways 40. Data from the Cancer Genome Atlas (TCGA) shows substantial CNPY2 upregulation in NSCLC subtypes, including lung squamous cell carcinoma (LUSC), and lung adenocarcinoma (LADC) correlating with poor prognosis 5.

**Figure 3:**
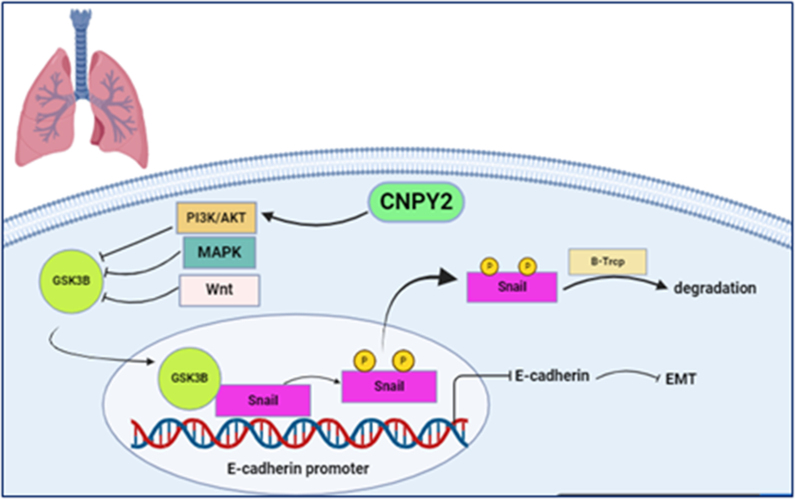
The role of CNPY2 in inducing epithelial-mesenchymal transition (EMT) in lung cancer through regulating the AKT/ GSK-3 β signaling pathway. CNPY2 overexpression stimulates AKT signaling, inhibits GSK-3 β , a kinase responsible for Snail phosphorylation and degradation. Inhibition of GSK-3 β stabilizes Snail and prevents its nuclear export, and subsequent degradation by β -Trcp. Stabilized Snail reduces E-cadherin expression and enhances expression of mesenchymal markers such as Vimentin. This molecular cascade restrains cell-cell adhesion and initiates EMT, a critical process in cancer cell invasion and metastasis. The figure was created with BioRender.com (https://www.biorender.com/).

One of the principal oncogenic activities of CNPY2 in NSCLC is its role in chemoresistance by triggering NF- κ B pathway, which interacts with the UPR pathway to enhance resistance to chemotherapy 41. Specifically, CNPY2 increases phosphorylation of I κ B α and IKK, facilitating NF- κ B nuclear translocation and upregulating anti-apoptotic proteins (c-IAP1, c-IAP2, XIAP, Bcl-2, and Bcl-xl), thereby reducing cisplatin-induced apoptosis. Conversely, CNPY2 knockdown or NF- κ B inhibition restores chemosensitivity 1. Beyond chemoresistance, CNPY2 drives NSCLC metastasis by promoting epithelial-mesenchymal transition (EMT), via the AKT/GSK3 β pathway 42. Mechanistically, CNPY2 overexpression activates AKT phosphorylation, inactivates GSK3 β and stabilizes Snail, a transcription repressor of E-cadherin, increasing tumor cell motility and invasiveness (Figure 3) 5. This cascade improves tumor cell motility and invasiveness, contributing to metastasis. Experimental inhibition of AKT signaling reverses EMT phenotypes in CNPY2-upregulated cells, underscoring its role in metastasis 5. These observations hopefully could spur further research to fully understand CNPY2’s role in the EMT phenotype, Snail stability, and in vivo tumor transformation 5. In the same note, studies also underline the regulatory influence of microRNA on CNPY2 expression in NSCLC. The miR-30a-3p/CNPY2 axis has been diagnosed as a critical regulator of EMT in LADC, where miR-30a-3p suppresses CNPY2 expression, reducing N-cadherin and increasing E-cadherin expression, suggesting a promising therapeutic target 43. These findings disclose CNPY2’s multifaceted roles in NSCLC and provide promising therapeutic opportunities for targeting CNPY2 to improve treatment outcomes.

### CNPY2 in renal cell carcinoma: A tissue- specific regulator of the P53 pathway

Accumulating evidence demonstrates the role of ER homeostasis in renal cancer carcinoma (RCC) onset and progression, yet CNPY2’s role remains poorly understood 44. Recent studies demonstrate that CNPY2 promotes RCC progression via enhancing TP53 pathway activity. Taniguchi *et al.* showed that simultaneous knockdown of both P53 and CNPY2 in 786-O cells significantly reduces CDKN1A expression, a key P53 target gene, validating CNPY2’s role in enhancing P53 transcriptional activity and contributing to RCC tumor growth 45. Tissue analyses further reveal a robust positive correlation between CNPY2 and P53 expression in RCC patients, reinforcing their functional interaction 46. Mechanistically, CNPY2 may upregulates P53 mRNA by modulating transcription factors such as RBP-J κ (a repressor) and C/EBP β -2 (an activator) 47, 48, while inhibiting MYLIP, as an E3 ubiquitin ligase, to prevent P53 degradation .

Tumor-specific factors, such as altered cofactors or HIF-1 α interactions, may shift P53 from tumor suppression to supporting RCC cell survival, potentially *via* post-translational modification such as phosphorylation and acetylation. Interestingly, CNPY2’s role is tissue-specific, it negatively regulates P53 to promote tumor growth in colorectal cancer but modulates P53 to support tissue repair in cardiac transgenic mice post-heart injury 49. These context-dependent CNPY2-P53 interactions, distinct from MDM2-mediated P53 regulation, underscore the need for further research to clarify RCC-specific mechanisms 50. By delving deeper into these interactions may, we could uncover new therapeutic opportunities targeting the CNPY2-P53 axis in RCC.

**Figure 4:**
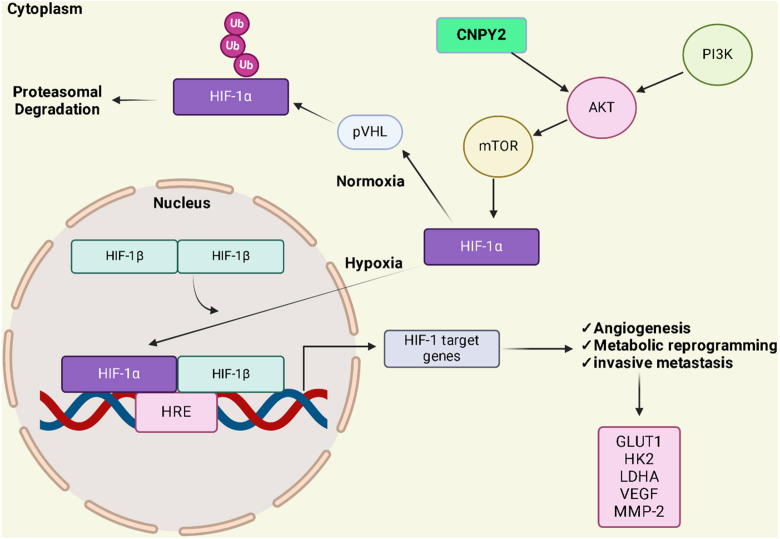
CNPY2 enhances hypoxia-induced glycolysis by the PI3K/Akt/mTOR pathway. Under normoxia, HIF-1 α is degraded via proline hydroxylases (PHDs) and von Hippel-Lindau protein (pVHL) activity, which facilitates its polyubiquitination and subsequent proteasomal degradation. CNPY2 overexpression activates the PI3K/Akt/mTOR pathway, stabilizing HIF-1 α , which translocate to the nucleus, dimerizes with HIF-1 β , binds to hypoxia-responsive elements (HREs), and upregulates genes involved in angiogenesis, metabolism and metastasis including glucose transporters (GLUTs), vascular endothelial growth factors (VEGFs), and matrix metalloproteinases (MMPs). AKT1, the serine-threonine protein kinase AKT1; GLUT-1, facultative glucose transporter 1; HIF, hypoxia-inducible factor; PI3K, phosphoinositide 3-kinase; Ub, ubiquitin; VEGF, vascular endothelial growth factor; VHL, von Hippel–Lindau protein. The figure was created with BioRender.com (https://www.biorender.com/).

### CNPY2 is transcriptionally regulated by HIF-1a and its role in cervical cancer

CNPY2, a transcriptional target of hypoxia-inducible factor 1 α (HIF-1 α ), plays a key role in cervical cancer progression by promoting cell proliferation, glycolysis, tumor survival under hypoxic conditions, correlating with poor prognosis 51–53. CNPY2 overexpression drives the Warburg effect (aerobic glycolysis), a metabolic shift to promote glucose consumption, and lactate production which supports tumor energy metabolism and angiogenesis (Figure 4) 54. Tian *et al.* found that CNPY2 knockdown significantly impairs glucose uptake, lactate production, and expression of glycolysis-related genes such as lactate dehydrogenase A (LDHA), hexokinase 2 (HK2), and glucose transporter 1 (GLUT1), thereby reducing cervical cancer cell viability 54. Furthermore, CNPY2 silencing attenuates the AKT pathway, a crucial hypoxia-activated pathway, attenuating tumor growth 54.

Under hypoxic conditions, HIF-1 α binds to CNPY2 promoter, upregulating its expression and promoting angiogenesis *via* enhancing smooth muscle cell proliferation and migration which helps tumor cells evade apoptosis 55. By inducing glycolytic enzymes, CNPY2 acidifies the tumor microenvironment, further enhancing HIF-1 α activity and hypoxia-mediated tumor growth 55. CNPY2 knockdown suppresses glycolysis and the hypoxic tumor microenvironment, highlighting its therapeutic potential 55. These evidences position CNPY2 as a principal regulator of hypoxia-driven metabolic reprogramming in cervical cancer, offering a promising diagnostic marker and therapeutic target 56.

### The role of CNPY2 in urothelial carcinoma and polycomb repressive complex 2 regulation

Genomic analyses identify CNPY2 as a potential regulator of polycomb repressive complex 2 (PRC2), a key epigenetic modulator involved in transcriptional repression 57. In non-muscle invasive bladder cancer (NMIBC), Huang *et al.* indicated a negative correlation between CNPY2 expression and H3K27me3 levels, a hallmark of PRC2-mediated gene silencing 57. Owing to elevated H3K27me3 levels are associated with advanced stages of upper tract urothelial carcinoma (UTUC), CNPY2 likely influences the epigenetic landscape of UC progression.

Although the precise functional mechanisms remain enigmatic, higher CNPY2 expression correlates with PRC2-mediated transcription regulation, potentially affecting tumor recurrence and progression risk stratification in bladder cancer patients. Further investigations are needed to delve deeper into CNPY2’s effect on PRC2 dynamics and its therapeutic potential in UC.

### Prognostic value of CNPY2 in HCV-related HCC and liver cancer progression

ER stress and UPR activation contribute to hepatocellular carcinoma (HCC), a leading cause of cancer-related mortality worldwide 58, 59. CNPY2’s role in HCC is well documented particularly in cases associated with NAFLD, and hepatitis C virus (HCV) 4, 25. Clinically, CNPY2 overexpression in HCV-positive HCC patients correlates with advanced tumor stage, poor differentiation, and reduced overall survival, emphasizing its potential as a prognostic biomarker and therapeutic target 60. Proteomic and transcriptomic investigation by Kakehashi *et al.* corroborates CNPY2’s oncogenic role, promoting focal adhesion, endocytosis and downregulation of growth factors signaling including FGF21 and HGF 60. Knockout models further support its role in regulation of survival and proliferation pathways via c-Jun, Fos, STAT5, Notch receptor 1, and VEGF, driving tumor progression 4, 60.

**Figure 5:**
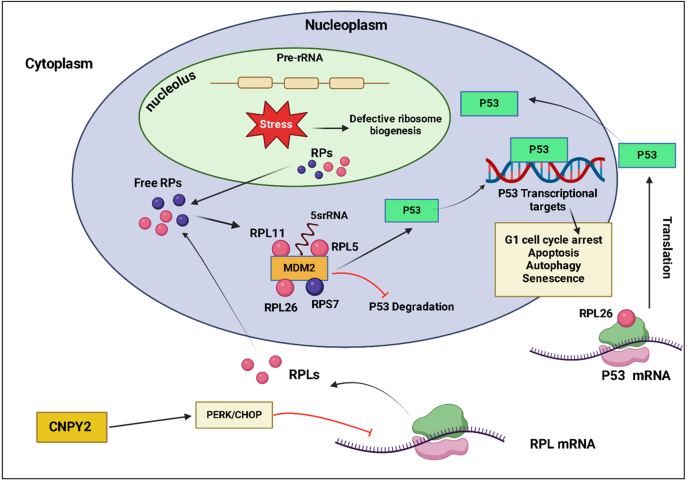
Mechanistic role of CNPY2 in P53 activation during cellular stress response. During cellular stress, ribosomal proteins (RPL5, RPL11), along with 5S ribosomal RNA (rRNA), are released into nucleoplasm. These components inhibit the activity of MDM2 ubiquitin ligase, resulting in the stabilization and accumulation of P53. Concurrently, in the cytoplasm, ribosome-free RPL26 binds to 5’ untranslated region (UTR) of p53 mRNA, inducing its translation. CNPY2 has a prominent role in the cellular stress response and tumor-suppressive function. It suppresses MDM2 activity through disrupting ribosome biogenesis and promotes the assembly of the RPL5-RPL11-5S rRNA complex. P53 in stabilize position upregulates its downstream genes that orchestrate vital cellular pathways including apoptosis, cycle arrest, and senescence. The figure was created with BioRender.com (https://www.biorender.com/).

Mechanistically, CNPY2 promotes hepatocellular carcinoma progression *via* regulating PERK and IRE1 pathways, promoting UPR signaling, supporting tumor cell survival. It also drives cell cycle progression and invasion *via* cyclin D1 and P21 (Cip/WAF1) regulation 12. In HCC cell lines with CNPY2 knockdown, UPR markers (phosphorylated eIF2 α , and CHOP) substantially decreases survival proteins (Bcl-2), and induces apoptosis resulting in a less aggressive cancer phenotype 12. CNPY2 also modulates MDM2-P53 axis, promoting P53 degradation via GSK3 β /MDM2 signaling 4. CNPY2 deletion stabilizes P53, upregulates ribosomal proteins (RPL5, RPL11) and 5S rRNA (ribosomal RNA), inhibits MDM2-mediated degradation, and induces cell cycle arrest and tumor suppression (Figure 5) 61, 62. In a 2025 study, CNPY2 knockdown in macrophages in a diethylnitrosamine (DEN)-induced liver cancer model, diminishes cytokine production and hepatocarcinogenesis, highlighting CNPY2’s role in inflammation and tumor development 63. These findings underscore CNPY2’s potential as both prognostic biomarker and therapeutic target in HCC, warranting further investigation to develop novel therapeutic strategies for liver cancer.

### The role of CNPY2 in colorectal cancer: Diagnostic, prognostic, and therapeutic implications

CRC, the third most common cancer and second leading cause of cancer-related deaths worldwide, underscore the need for early detection to decrease mortality rates 64. CNPY2 isoform 2 is an emerging biomarker with both diagnostic and prognostic significance in CRC 15. Investigations in HTC116 cells and xenograft models demonstrate that CNPY2 knockdown enhances P53 activity, subsequently increasing p21 expression, decreasing cyclin-dependent kinase 2 (CDK2), and inhibiting tumor growth, migration and angiogenesis 12. CNPY2 knockdown also remarkably enhances oxaliplatin sensitivity by activating caspase-3 and -8 and elevating the ratio of phosphorylated p53 at Ser15 to total p53, indicating CNPY2’s role in chemoresistance *via* inhibition of intrinsic and extrinsic apoptosis pathways 12. An investigation by Peng *et al.* confirmed that higher CNPY2 isoform 2 expressions in CRC tissues compared to normal colonic tissues and epithelium, with lower expression correlated with worse 5-year overall survival (OS) 15. In 2018, they identified CNPY2 as an independent prognostic marker, combining it with carcinoembryonic antigen (CEA) and CA 19–9 improved diagnostic accuracy across all stages of CRC 7. Moreover, they confirmed that the diagnostic efficacy of these three markers profoundly enhances compared to utilizing each marker individually in CRC detection 7. Hence, incorporating CNPY2 into non-invasive tests such as Guaiac-based fecal occult blood tests (GFOBT) and fecal immunochemical tests (FITs), could enhance early CRC detection 7.

Additionally, the saposin B-like domain of CNPY2 isoform 2 may influence immune responses by facilitating CD1d-mediated antigen presentation, activating natural killer (NKTs) cells and enhancing CD8+ T cell responses 15, 65. There is a strong consensus that both NKT cells and CD8+ T cells are vital components for antitumor immunity, low CNPY2 isoform 2 expression may impair these immune responses, worsening survival outcomes 15, 66, 67.

Further research is required to clarify the CNPY2 isoform 2’s molecular mechanisms and therapeutic potentials. It would be helpful to experimentally interrogate isoform-specific functions, *via* clone and express tagged isoform variants to assess localization, stability, and binding to known CNPY2 partners such as MYLIP, P53. Moreover, we can suggest performing isoform-selective knockdown or overexpression in cell lines or xenograft models to evaluate effects on proliferation, migration, signaling and immune interactions. Clinically, CNPY2 could utilize as a supplementary diagnostic tool, with low-expression patients potentially benefiting from intensified postoperative monitoring and chemotherapy.

### CNPY2 as a prognostic biomarker in esophageal squamous cell carcinoma (ESCC)

Esophageal squamous cell carcinoma (ESCC) is a highly lethal malignancy worldwide 68. Recent studies identify CNPY2, alongside protein disulfide isomerase family A member 3 (PDIA3), and stathmin 1(STMN1), as a key prognostic factor in ESCC, with five-year overall survival and disease-free survival (DFS) predictive accuracies of 84.6% and 72.6%, respectively 69. Mass spectrometry analyses reveal CNPY2 as an interacting partner of Ezrin, a cytoskeletal protein that regulates tumor invasion and metastasis 69. High CNPY2 expression correlates with increased lymph node metastasis, poor patient prognosis, and substantially reduced overall and recurrence-free survival (RFS) in ESCC patients 69. Given its prognostic potential, CNPY2 also could serve as a precious biomarker and therapeutic target, though, its precise mechanisms in ESCC pathogenesis remain enigmatic and warrant further research.

## BENEFITS OF CNPY2/MIRNAS PATHWAY AS A POTENTIAL THERAPEUTIC TARGET IN CANCER

Targeting the CNPY2/miRNAs pathway represents a promising therapeutic avenue to overcome treatment resistance and improve cancer prognosis, in LADC, gastric cancer and glioma 43, 70–72. Emerging research emphasizes its potential address treatment resistance and improves cancer prognosis.

### miRNA-30e-3p/CNPY2 pathway and temozolomide (TMZ) sensitivity in glioma

Glioblastoma, as aggressive brain cancer, presents remarkable treatment challenges due to drug-resistance to temozolomide (TMZ), the first-line chemotherapy. Gao et.al reported that miR-30e-3p regulates TMZ sensitivity via downregulating CNPY2 expression, inhibitng glioma cell growth and migration while inducing apoptosis  72. CNPY2 overexpression also promotes DNA damage repair by reducing γ -H2AX levels, a marker of DNA double-strand breaks 72. Given the importance role of the miR-30e-3p/ CNPY2 axis, further research is needed to assess its prognostic value and explore additional regulatory mechanisms influencing this pathway 73.

### miR-30a-3p/CNPY2 interaction in lung adenocarcinoma (LADC)

In lung adenocarcinoma (LADC), CNPY2 is a target of the tumor suppressor miR-30a-3p. analysis of TCGA data by Wang *et al*. ascertained consistent CNPY2 upregulation in LADC, irrespective of clinical stage or patient demographics, with a negative correlation between miR-30a-3p and CNPY2 expression 43. Downregulation of miR-30a-3p increases CNPY2 expression, promoting cancer cell proliferation and migration by regulating EMT-associated proteins in LADC cells, including downregulation of N-cadherin and upregulation of E-cadherin. 43. Thus, miR-30a-3p overexpression or CNPY2 inhibition may represent an innovative therapeutic approach for LADC 43. Hight CNPY2 expression also associates with poor patient survival, emphasizing its potential as a diagnostic and prognostic marker, consistent with findings in other cancers 12, 46. Recent evidence confirms that CNPY2 mRNA selectively binds to miR-30a-3p, but not miR-30a-5p, further supporting the selective regulation by miR-30a-3p in LADC. Taken together, these studies highlight the therapeutic potential of targeting the miR-30a-3p/CNPY2 axis in LADC, alongside the miRNA-30e-3p/CNPY2 pathway in glioma, as promising avenues for cancer treatment 43.

### A novel LINC00342/miR-545-5p/CNPY2 regulatory axis in gastric cancer

The Long non-coding RNAs (lncRNAs) influence cancer pathogenesis by acting as competitive endogenous RNA (ceRNAs), regulating gene expression post-transcriptionally 74, 75. Liu *et al.* uncovered the LINC00342, and miR-545-5p/CNPY2 axis as a key regulator in gastric cancer (GC), highlighting its potential as therapeutic target. LINC00342 functions as a ceRNA, sequestering miR-545-5p to upregulate CNPY2 expression, thereby promoting tumor cell proliferation, colony formation, migration, and invasion 71. In vivo experiments confirmed that LINC00342 knockdown suppresses tumorigenesis, reduces CNPY2 expression and decreases Ki-67 positive tumor cells 71. These findings elucidate post-translational modulation of CNPY2 through lncRNAs, underscoring the therapeutic promise of targeting the LINC00342/ miR-545-5p/CNPY2 axis in GC.

## CNPY2 AS AN IMMUNE REGULATOR IN TUMOR PROGRESSION

CNPY2 plays a pivotal role in immune regulation within the tumor microenvironment (TME), driving cancer progression, immune invasion, and therapeutic resistance. It modulates tumor associated macrophages (TAM), ER stress, and hypoxia-induced angiogenesis, linking inflammation to tumor growth. A 2024 study reported that CNPY2 as a toll-like receptor 4 (TLR4) regulator, triggering cytokine production in macrophages 63. In macrophage-specific CNPY2 knockout (KO) mice, diethylnitrosamine (DEN)- induced HCC was remarkably diminished due to a decreased TLR4 surface expression, inhibiting NF- κ B nuclear translocation and proinflammatory cytokines production (IL-6, IL-23), while anti-tumoral M1 macrophages remained unaffected, proposing a dramatic shift towards an anti-inflammatory macrophage profile 63. RNA sequencing disclosed lower mRNA and surface expression of VEGF receptors (Flt1, kdr) in CNPY2 KO macrophages, limiting macrophage recruitment to tumors by reducing NF- κ B/P52-mediated transcription of these receptors 63.

Beyond macrophages, CNPY2 could also suggest induces ER stress-related T cell dysfunction, where prolonged UPR signaling contributes to T cell exhaustion, impairing cytotoxic responses 76. Preclinical studies suggest combining CNPY2 with immune checkpoint inhibitors (anti-PD-1, anti-CTLA-4), to enhance outcomes across cancers 76. We are still in the early stage of understanding the role of CNPY2 in immunology concepts with the hope to gain more insights for transforming into clinical fields.

## CNPY2 ROLE IN NON-CANCER DISEASE

### CNPY2 implication in cardiovascular homeostasis and ischemia-reperfusion injury

Emerging evidence underscores CNPY2’s critical role in cardiovascular homeostasis, principally by stabilizing compensatory hypertrophy and inducing angiogenesis 8, 49. CNPY2 is stringently regulated by hypoxia-inducible factor 1-alpha (HIF-1 α ), a key driver of ischemic cardiac angiogenesis 77–79. In response to hypoxia-induced ischemic injury, CNPY2 upregulates HIF-1 α and vascular endothelial growth factor (VEGF), enhancing revascularization, smooth muscle cell migration and proliferation 14, 49, 80. Enigmatically, CNPY2 triggers the PERK pathway in endothelial cells, linking the UPR to cardiovascular repair 81. Evidence further demonstrate that the glucagon-like peptide-1 receptor agonist Liraglutide (Lir) increases HIF-1 α , and VEGF expression *via* a CNPY2-PERK dependent pathway in hypoxia/reoxygenation (H/R) injury models, underscoring the therapeutic potential of this axis 81. *In vivo*, elevated CNPY2 levels in nucleophosmin 1 (Npm1)-conditional knockout (cKo) mice post-myocardial infarction (MI), correlate with reduced inflammation, promoted angiogenesis and improved mitochondrial function, ultimately leading to myocardial protection and cardiac recovery. Notably, CNPY2 synergizes with growth differentiation factor 15 (GDF15) to support cellular growth and tissue repair, indicating that NPM1 inhibition in macrophages may offer a therapeutic strategy for heart recovery 77. Conversely, CNPY2 silencing impairs post-myocardial infarction recovery through upregulating the p16INK4/cyclin D1/Rb pathway and decreasing PDK1/Akt activity, leading to reduced cardiac repair and functional recovery 78.

Beyond its role in cardiac repair, CNPY2 contributes to the pathogenesis of hypertension. Mechanistically, angiotensin II remarkably upregulates CNPY2 in murine and human endothelial cells, activating the PERK/eIF-2 α /CHOP signaling pathway. This activation elevates cytoplasmic Ca ${}^{2+}$ levels, promotes phosphorylation of CaMKII (Thr286), and induces Drp1-dependent mitochondrial fission. Conversely, CNPY2 knockdown mitigates these effects, highlighting its potential as a therapeutic target in hypertension 79.

CNPY2 also contributes to ischemia-reperfusion injuries including spinal cord (SCIRI), cerebral (CIRI), and myocardial (MIRI) injuries. In SCIRI, CNPY2-PERK activation promotes neuronal apoptosis, which dexmedetomidine (Dex), an $\alpha_{2}$ -adrenergic agonist alleviates 82. Similarly, in CIRI, a potential bioactive agent berberine (BBR) downregulates the CNPY2-PERK pathway, reducing apoptotic protein and providing neuroprotection 83. In MIRI, barbaloin, a naturally arising bioactive anthracycline, protects cardiomyocytes *via* suppressing CNPY2-PERK mediated apoptosis, decreasing GRP78 and caspase-12, and inhibiting cardiomyocytes apoptosis 84.

Collectively, these findings propose that targeting CNPY2 could offer a new therapeutic approach for moderating ischemia-reperfusion injuries across multiple organs. Given its role in cardiac recovery and hypertension, further investigation is required to fully elucidate CNPY2-targetd therapies in clinical settings.

### CNPY2 and genetic susceptibility in Myasthenia gravis

Myasthenia gravis (MG), a common neurological autoimmune disease is characterized by autoantibodies targeting postsynaptic antigens at the neuromuscular junction 85. Recent genetic studies link MG susceptibility to CNPY2, with the HLAB08:01 allele and rs10783780, a single nucleotide polymorphism (SNPs) downstream of CNPY2 gene, associated with increased risk 86. Although rs10783780 does not directly correlate with CNPY2 mRNA levels, MG patients exhibit significantly lower CNPY2 expression, suggesting the CNPY2 gene cluster may serve as a susceptibility locus, exclusively in patients with the HLAB08:01 allele 86. Additionally, linkage disequilibrium (LD) between polymorphisms in the citrate synthase (CS) and CNPY2 genes illustrates potential co-regulation, requiring further investigation to clarify the functional aspects of CNPY2 role in MG progression 86.

### Neuroprotective role of CNPY2 in Parkinson’s disease

Parkinson’s disease (PD), a neurodegenerative disorder characterized by progressive dopaminergic neurons loss, is mitigated by CNPY2’s neuroprotective effects via activation of the AKT/ GSK3 β signaling pathway 87. Mechanistically in PD mouse models, CNPY2 overexpression, reduces oxidative stress, mitochondrial dysfunction, and neuronal apoptosis by reducing pro-apoptotic proteins (*e.g.*, Bax, cleaved caspase-3), and increasing anti-apoptotic Bcl-2 while promoting neurite growth. Pharmacological inhibition of AKT with MK-2206 2HCl reverses these effects, validating the AKT/ GSK3 β pathway’s role in CNPY2- mediated neuroprotective 87. These observations position CNPY2 as a promising therapeutic target for preserving dopaminergic neurons in PD, warranting further investigations to elucidate its signaling mechanisms and therapeutic potential in disease progression and intervention.

### The CNPY2-CTIP2 interaction in Huntington’s disease: A potential therapeutic avenue

Huntington’s disease (HD), an dominant neurodegenerative disorder initiated by CAG expansion in exon 1 of the huntingtin gene, leads to corticostriatal circuits degeneration 88, 89. CNPY2 is upregulated in striatal neurons expressing mutant huntingtin, regulating ER stress *via* the IRE1 α -XBP1s-GRP78 axis. Interestingly, CNPY2 may act extracellularly on neurons, though its mechanisms warrant further investigations 90. In Human neuroblastoma SH-SY5Y cells, CNPY2 overexpression enhances cell viability under tunicamycin- induced ER stress, whereas knockdown exacerbates neuronal loss 90. Furthermore, CNPY2 interacts with COUPTF-interacting protein 2 (CTIP2/BCL11B), a crucial transcription factor for cortical and striatal neurons survival, proposing a neuroprotective role 91, 92. Given CNPY2’s dual modulation of ER stress and neuronal survival, further studies are needed to characterize downstream targets and evaluate its therapeutic potential in HD.

### CNPY2 as a promising target in keratoconus disease

Keratoconus (KC), a progressive, bilateral and asymmetric ocular disease is characterized by corneal thinning, leading to irregular astigmatism and myopia 93–95. CNPY2 is significantly downregulated in KC and is a key player in its pathogenesis 96. A systematic meta-analysis by Song *et al.* identified 202 differentially expressed proteins in KC patients compared to healthy controls, with CNPY2 among the top 10 down-regulated proteins 96. According to Gene Ontology (GO) and Kyoto Encyclopedia of Genes and Genomes (KEGG) analyses revealed that these proteins are involved in inflammation, extracellular matrix (ECM) remodeling, and apoptosis, hallmark processes of keratoconus, primarily linked to the IL-17, TNF, MAPK and TGF-beta signaling pathways 96. Future research on CNPY2 and related proteins could uncover novel biomarkers and therapeutic target for KC.

## CONCLUSION AND FUTURE DIRECTIONS

CNPY2 is a pivotal regulator of ER stress and UPR, with implications for cancer, neurological disorders, and cardiovascular diseases. This review consolidates current knowledge into CNPY2’s interactions with P53, MYLIP-mediated androgen receptor regulation, NF- κ B, and AKT/GSK3 β pathways, alongside its multifaceted roles in the tumor microenvironment, including hypoxic responses, angiogenesis, and immune modulation. Its effects on P53 and related signaling seem determined by the status of key pathways components, tumor microenvironment, resulting in either pro-growth, pro-survival, or anti-apoptotic outputs. Thus, CNPY2 acts as a pleiotropic signal integrator, and its biological consequence in cancer is highly context and tissue-specific, demonstrating the general principle that adaptive regulators operate differently across heterogenous tumor systems. Clinically, CNPY2 serves as a non-invasive diagnostic and prognostic marker, with elevated tumor or serum CNPY2 levels correlating with worse overall survival and unfavorable prognoses in liver, colorectal, and non-small lung cancers. Hence, integrating CNPY2 testing into routine clinical approaches could promote diagnostic algorithms, specifically when histopathological results are inconclusive, potentially decreasing diagnostic delays and enhancing patient outcomes.

Regardless of remarkable progress in elucidating CNPY2’s molecular functions, several key gaps persist, highlighting priorities for future investigation. Isoform and mechanism-specific studies are required to define its regulatory mechanisms, isoform-specific roles, and broader implications in disease pathogenesis. High-resolution structural approaches, such as X-ray crystallography and cryo-electron microscopy, could reveal the structural basis of CNPY2’s interaction with PERK and MYLIP. Animal and cell-based studies have recognized a strong link between CNPY2 expression and chemotherapy resistance *via* oncogenic and UPR pathways. Understanding how CNPY2 impacts tumor microenvironment, and the immune system could unlock new immunotherapeutic opportunities for inflammation-driven oncogenesis. Advanced techniques, including single-cell RNA sequencing and proteomic profiling, should be employed to investigate CNPY2’s immunomodulatory roles, precisely in inflammation and TAMs regulation, and metabolic reprogramming.

Elucidating CNPY2’s interactions with non-coding RNAs, such as miR-30a-3p in lung adenocarcinoma and miRNA-30e-3P in glioma, could uncover promising therapeutic opportunities. Advances in targeted drug delivery systems, including small-molecule inhibitors, monoclonal antibodies, and nanoparticle-based therapies, may enhance therapeutic precision and reduce off-target effects. To gain deeper mechanistic insights, future studies should leverage large-scale multi-omics approaches, proteomics, transcriptomics, and metabolomics in vivo models, focusing on disease-specific contexts. Moreover, investigating CNPY2’s effect on metabolic signaling pathways and developing selective inhibitors or activators could further clarify its role in various pathological conditions. Overall, CNPY2 is a pleiotropic, context-dependent stress adaptive regulator whose isoform, provide various opportunities for therapeutic exploration.

## AUTHORS CONTRIBUTIONS

SE: drafting of the manuscript; LM: revision of the manuscript; FH: concept and design; supervision; critical revision of the manuscript.

## CONFLICT OF INTEREST

The authors declare no competing interests.

## ABBREVIATIONS

AR – androgen receptor

ceRNA – competitive endogenous RNA

CRC – colorectal cancer

CNPYs – canopy homolog proteins

EMT – epithelial-mesenchymal transition

ER – endoplasmic reticulum

ESCC – esophageal squamous cell carcinoma

GC – gastric cancer

GLUT – glucose transporter

HCC – hepatocellular carcinoma

HD – Huntington’s disease

HIF – hypoxia-inducible factor

IDOL – inducible degrader of the LDL receptor

KC – Keratoconus

LADC – lung adenocarcinoma

LDLR – low-density lipoprotein-cholesterol receptor

lncRNAs – long non-coding RNAs

MYLIP – myosin regulatory light chain interacting protein

NSCLC – non-small-cell lung cancer

PD – Parkinson’s disease

RCC – renal cancer carcinoma

SAPLIP – saposin-like protein

TAMs – tumor associated macrophages

TCGA – the Cancer Genome Atlas

TLR – Toll-like receptor

TMZ – temozolomide

UC – urothelial carcinoma

UPR – unfolded protein response

VEGF – vascular endothelial growth factor
